# The effect of the amount of blocking cue training on blocking of appetitive conditioning in mice

**DOI:** 10.1016/j.beproc.2015.11.007

**Published:** 2016-01

**Authors:** David J. Sanderson, William S. Jones, Joseph M. Austen

**Affiliations:** Department of Psychology, Durham University, South Road, Science Site, Durham, DH1 3LE, UK

**Keywords:** Blocking, Conditioning, Learning, Memory, Mice

## Abstract

Conditioning of a target cue is blocked when it occurs in compound with another cue (blocking cue) that has already received conditioning. Although blocking of appetitive conditioning is commonly used in rodents as a test of selective learning, it has been demonstrated rarely in mice. In order to investigate the conditions that result in blocking in mice two studies tested the effect of the extent of prior blocking cue training on blocking of appetitive conditioning. Mice received either 80 or 200 trials of blocking cue training prior to compound conditioning. A control group received only compound training. Experiment 1 assessed the ability of a visual cue to block conditioning to an auditory target cue. Exposure to the context and the unconditioned stimulus, sucrose pellets, was equated across groups. Blocking was evident in mice that received 200, but not 80 training trials with the visual blocking cue. Responding to the blocking cue was similar across groups. Experiment 2 assessed the ability of an auditory cue to block conditioning to a visual target cue. Blocking was evident in mice trained with 80 and 200 auditory blocking cue trials. The results demonstrate that the strength of blocking in mice is dependent on the modality and experience of the blocking cue. Furthermore, prolonged training of the blocking cue after asymptotic levels of conditioned responding have been reached is necessary for blocking to occur under certain conditions suggesting that the strength of conditioned responding is a limited measure of learning.

## Highlights

• Blocking of appetitive conditioning in mice has rarely been demonstrated.
• Blocking occurred when there was 200, but not 80 trials with a visual blocking cue.
• Blocking occurred independent of trial number with an auditory blocking cue.
• Post-asymptotic training is necessary under certain conditions for blocking.

## 1 Introduction

In a conditioning procedure a cue that has been paired with an unconditioned stimulus (US) may fail to elicit conditioned responding if the cue has been conditioned in compound with another cue that has previously been paired with the unconditioned stimulus (Kamin, 1969). This blocking effect provides an example of the failure of temporal contiguity between events to be sufficient for conditioning. It also demonstrates that there is competition between cues that may reflect selective learning, through changes in processing of the unconditioned stimulus (Rescorla and Wagner, 1972) or attention paid to the different conditioned stimuli (Pearce and Hall, 1980, Mackintosh, 1975). Alternatively it may reflect a failure to behaviourally express learning (Stout and Miller, 2007).

Blocking has been demonstrated in numerous species and conditioning procedures (e.g., fear conditioning in rats, Kamin, 1969; autoshaping in pigeons, Leyland and Mackintosh, 1978; odour conditioning in snails, Prados et al., 2013; conditioning of the nictitating membrane response in rabbits, Solomon, 1977; electrodermal conditioning in humans, Hinchy et al., 1995). However, surprisingly, considering the widespread use of mice for assessing the neural basis of learning, there are few examples of blocking in mice (Bonardi et al., 2010). One of the most common ways of assessing learning in rodents is by appetitive conditioning of magazine approach behaviour, in which pairing a cue with food reward (e.g., a sucrose pellet) results in rodents making anticipatory head entries, during the conditioned stimulus (CS), into the magazine, where food is dispensed. To our knowledge, a study by Bonardi et al. (2010) is the only study reporting blocking of appetitive conditioning in mice. In that study mice received 90 conditioning trials with a 20 s light before receiving conditioning with a compound of the light and a clicker stimulus. A control group received only training with the clicker and light compound. At test mice that received the light conditioning trials showed lower levels of magazine approach behaviour to the clicker compared to the control group.

Given the scarcity of evidence for blocking in mice the aim of the present study was to extend the findings of Bonardi et al. (2010) by examining one of the key parameters in determining whether a conditioning procedure will yield a blocking effect. In two experiments the number of conditioning trials with the blocking cue, prior to compound conditioning, was manipulated. Many theories of learning assume that the extent of blocking will be a function of the number of blocking cue conditioning trials (e.g., Rescorla and Wagner, 1972), and that blocking will be maximal if the blocking cue has acquired the maximum level of associative strength. However, while models of learning such as Rescorla and Wagner (1972) assume a direct relationship between associative strength and performance (e.g., asymptotic levels of conditioned responding indicate maximum levels of learning), it is likely the case that conditioned responding is not a pure measure of learning. For example, by using particular probe tests it is possible to demonstrate that levels of learning continue to increase beyond the point that asymptotic levels of conditioned responding have been achieved (St. Claire-Smith and Mackintosh, 1974). In the study by St. Claire-Smith and Mackintosh (1974) it was found that although a compound of a tone and a light elicited asymptotic levels of conditioned fear, further pairings of the compound with shock increased the conditioned response to the tone and light when each stimulus was tested separately. Such dissociations between performance and learning have been found in other circumstances. For example, Holland and Rescorla (1975) trained a light to predict the occurrence of food (first-order conditioning) and then subsequently paired a clicker with the light (second-order conditioning). It was found that the clicker produced greater conditioned responding than the light despite the fact that it had not been paired with food. Therefore, although the light was not capable of eliciting strong conditioned responding itself, it had acquired a substantial amount of associative strength to support second-order conditioning of the clicker. Other studies have also found similar dissociations that suggest the strength of the conditioned response fails to convey the information that is acquired by the CS. For example, a backward conditioned cue elicits poor conditioned responding, but is a more effective second-order conditioning cue than a forward conditioned stimulus (Barnet et al., 1997). Similarly a trace conditioned stimulus that elicits weak responding can support stronger second-order conditioning than a cue with a shorter CS-US interval (Lin and Honey, 2011). These results demonstrate that the level of conditioned responding elicited by a cue is potentially a poor index of learning.

In the present study mice either received 80 trials or 200 trials of training with the blocking cue prior to compound conditioning. We have previously observed (in unpublished studies) that 80 trials with a 10 s CS (10 trials per daily session with an inter-trial interval of 240 s, CS offset to CS onset) typically yields asymptotic levels of conditioned responding, regardless of the modality (visual or auditory) of the CS. Therefore, for the mice that received 200 trials conditioning should continue substantially past the amount of trials sufficient to elicit maximum performance. In Experiment 1 we tested the ability of a visual cue to block conditioning of an auditory cue, similar to the procedure used by Bonardi et al. (2010). In Experiment 2 we tested the ability of an auditory cue to block conditioning of a visual cue. Given that there is evidence showing that auditory cues elicit greater levels of magazine activity than visual cues (Holland, 1977) it is possible that the parameters that determine blocking may differ when a visual cue is used to block an auditory cue and vice versa.

## 2 Method

### 2.1 Subjects

Experimentally naive female C57BL/6J/Ola mice obtained from Charles River, UK were used. Mice were caged in groups of four, in a temperature controlled housing room (light–dark cycle: 0800–2000). The mice were approximately 10 weeks old and a mean weight of 18.9 g (range = 16.8–21.4 g) at the start of testing. Mice were initially allowed free access to food, but one week prior to training the weights of the mice were reduced, by receiving a restricted diet, and then subsequently maintained at 85% of their free-feeding weights. Mice were tested during the light period between 10 am and 4 pm. Throughout testing mice had ad libitum access to water in their home cages. All procedures were in accordance with the United Kingdom Animals Scientific Procedures Act (1986), under project license number PPL 70/7785.

### 2.2 Apparatus

Eight identical operant chambers (15.9 × 14.0 × 12.7 cm; ENV-307A, Med Associates), enclosed in sound-attenuating cubicles (ENV-022 V, Med Associates), controlled by Med-PC IV software were used. The front and back walls and the ceiling of each chamber were made from clear Perspex and the sidewalls were made from aluminium. The floor was a grid of stainless steel rods (0.32 cm diameter) each separated by 0.79 cm. Sucrose pellets (14 mg TestDiet, ETH) could be dispensed into a magazine (2.9 × 2.5 × 1.9 cm; ENV-303 M, Med Associates) located in the centre of one of the sidewalls. Breaks in an infrared beam (ENV-303HDM, Med Associates) across the bottom of the entrance to the magazine were used to measure the number of magazine head entries at a resolution of 0.1 s. White noise (ENV-325SM, Med Associates) could be emitted from a speaker (ENV-324 M, Med Associates) located at the top right corner of the wall opposite the magazine. A clicker (ENV-335 M, Med Associates) was located on the exterior top left corner of the wall opposite the magazine. A 28 V, 100 mA house light (ENV-315 M, Med Associates) was located next to the speaker in the centre of the wall. Presentation of the house light resulted in illumination of the chamber. Two LEDs (ENV-321 M, Med Associates) were positioned to the left and the right, above the magazine. Presentation of the LEDs resulted in limited, localised illumination. A fan (ENV-025AC) was positioned above the left LED and was turned on during sessions.

### 2.3 Procedure

#### 2.3.1 Experiment 1—blocking of an auditory cue

Forty-eight mice were run in two separate cohorts (24 each). Mice in both cohorts were randomly allocated to one of three groups: Blocking-80 trials, Blocking-200 trials, Control. There were eight mice per group, per cohort. The design of Experiment 1 is described in Table 1.

**Stage 1.** Mice received 12 sessions (one per day) of training with a 10 s CS (either cue A or B) that terminated in the presentation of a sucrose pellet. Each session contained 10 trials, with a variable inter-trial interval (CS offset to CS onset) of 240 s (range = 126–516, based on a Fleshler–Hoffman distribution (Fleshler and Hoffman, 1962)). Group Blocking-200 trials received training with cue A, which was illumination of the house light. Groups Blocking-80 trials and Control received training with cue B. For the first cohort cue B was 10 s presentation of the clicker (2 clicks per second), and for the second cohort B was a 10 s presentation of flashing LEDs (1 s on/1 s off).

**Stage 2.** Mice received eight sessions of training that were identical to those in the previous stage, but now group Blocking-80 trials received training with cue A and not cue B.

**Stage 3.** All mice received eight sessions of AX compound training. Cue X was a 10 s presentation of white noise, and was presented simultaneously with cue A. All other details were the same as Stages 1 and 2.

**Test.** During the test phase mice received extinction test sessions with cues A and X that were interspersed with additional AX compound training sessions. Specifically, on session 29 mice received 10 nonreinforced presentations of X. On session 30 mice received 10 nonreinforced presentation of A. Sessions 31–32 were additional AX training sessions that were identical to those in Stage 3. Session 33 was an extinction test session with X, identical to session 29. Session 34 was an extinction test session with A, identical to session 30. All other details were the same as Stages 1–3.

#### 2.3.2 Experiment 2—blocking of a visual cue

Forty-eight mice were run in two separate cohorts. For the first cohort the procedure was the same as for Experiment 1, except that cue A was the white noise, cue B was the clicker and cue X was the house light. For the second cohort, the details were the same as the first cohort except that exposure to the context and the US was not equated between the groups. Therefore, group Blocking-80 trials and group Control received no training during Stage 1, and remained in their home cages for the duration of the stage. In Stage 2 group Control did not receive training, but now group Blocking-80 trials started training with cue A and group Blocking-200 trials continued training with A. In Stage 3 all groups commenced compound AX training. There were eight mice per group, per cohort. All other details were the same as Experiment 1.

### 2.4 Data and statistical analyses

The number of head entries into the magazine was recorded in 1 s time bins during the CSs and during the 10 s period prior to each CS presentation, and is expressed as responses per minute (RPM). Responding was converted to difference scores in which the rate of responding during the pre-CS period was subtracted from that of the CS. For analysis of the extinction tests responding during the course of the CS was examined in 2 s time bins. The average rate of responding during the pre-CS period was subtracted from the rate during the CS for each time bin. Data were assessed using multi-factorial analyses of variance. The Greenhouse-Geisser correction was used for violations of sphericity. Significant interactions were analysed using simple main effects analysis using the pooled error term from the original ANOVA, or separate ANOVAs for repeated measures with more than two levels. Significant between group effects were analysed using the Bonferroni correction for multiple comparisons.

## 3 Results

### 3.1 Experiment 1—blocking of an auditory cue

**Fig. 1:**
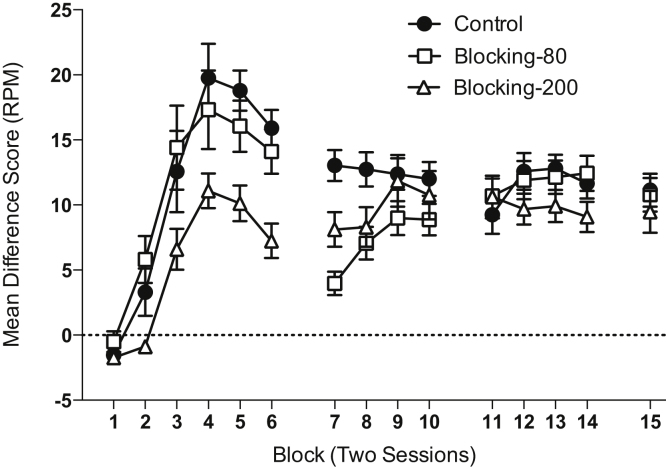
Performance during stages 1 (blocks 1–6), 2 (blocks 7–10) and 3 (blocks 11–15) of Experiment 1. The results of block 15 are separated from those of 11–14 due to the sessions being interleaved between the extinction test sessions. Responding is shown as difference scores in which the rate of magazine entries during the pre-CS period is subtracted from that during the CS. Error bars indicate ±SEM.

**Stage 1.** The results of Stage 1 are shown in Fig. 1. Responding was assessed in two session blocks of training. Responding increased over blocks of training and peaked at the forth block in all groups. There was a mild reduction in responding after the forth block. Group Blocking-200 trials that received training with cue A showed weaker levels of responding compared to groups Blocking-80 trials and Control that received training with cue B. A group by block ANOVA revealed a significant effect of block (*F*(5,225) = 77.15, *p* < 0.001), and a significant effect of group (*F*(2,45) = 6.05, *p* = 0.005). The interaction between factors was not significant (*F*(10,225) = 1.96, *p* = 0.083).

A similar analysis of the pre-CS levels of responding (overall mean = 2.85 RPM ±0.17 SEM) showed a significant effect of block (*F*(5,225) = 12.89, *p* < 0.001) that was due to a reduction of responding over training. There was no significant effect of group or interaction between factors (*F* values < 1, *p* values > 0.80).

**Stage 2.** Responding increased over blocks for group Blocking-80 trials, which commenced training with cue A (see Fig. 1). However, by the end of stage 2 performance converged between the three groups. There was a significant effect of block (*F*(3,135) = 6.86, *p* = 0.001) and group (*F*(2,45) = 4.79, *p* = 0.013) and a significant interaction of factors (*F*(6,135) = 3.66, *p* = 0.005). Simple main effects analysis of the interaction revealed that there was a significant effect of group on blocks 7 and 8 (*F*(2,45) values > 4.70, *p* values < 0.02), but not on the other blocks (*F* values < 1.50, *p* values > 0.20). Both the Blocking-80 trials and Blocking-200 trials group showed a significant effect of block (*F*(3,45) values > 4.90, *p* < 0.001), but the Control group did not (*F* < 1, *p* > 0.80). Analysis of the pre-CS levels of responding (overall mean = 1.50 RPM ±0.11 SEM) failed to reveal any significant main effects or interactions (*F* values < 3.10, *p* values > 0.05).

**Stage 3.** During compound training the performance of the three groups was similar and there were no significant effects or interactions of factors (*F* values < 1.50, *p* values > 0.20, see Fig. 1). Analysis of the pre-CS levels of responding (overall mean = 1.21 RPM ±0.08 SEM) showed a significant effect of block (*F*(4,180) = 8.71, *p* < 0.001), reflecting a continued decline of responding over training. There was no significant effect of group (*F*(2,45) = 1.87, *p* = 0.17) and no significant interaction of factors (*F*(8,180) = 1.25, *p* = 0.29).

**Fig. 2:**
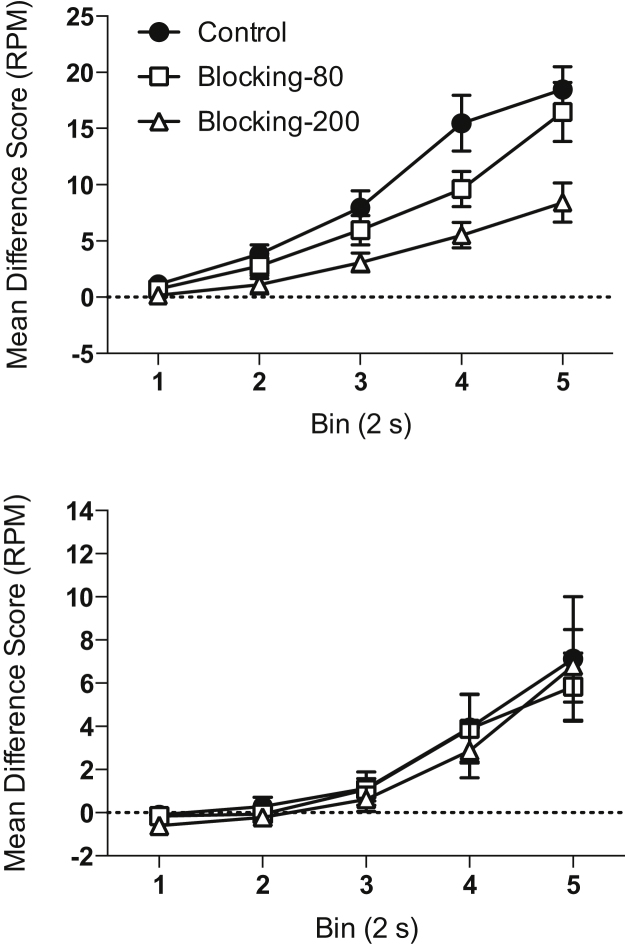
Test performance in Experiment 1. Conditioned responding during the blocked cue (top panel) and blocking cue (lower panel) is shown as difference scores (rate of magazine entries during CS minus the rate of magazine entries during pre-CS period) in five 2 s bins. Error bars indicate ±SEM.

**Test stage.** Blocked cue. Responding to the blocked cue during the Test Stage is shown in Fig. 2, upper panel. Responding during the presentation of the blocked cue (X) was analysed in five 2-second time bins. For all groups, responding increased over the duration of the CS. However, responding towards the end of the CS was higher for groups Control and Blocking-80 trials than for group Blocking-200 trials. There was a significant effect of bin (*F*(4,180) = 75.64, *p* < 0.001) and group (*F*(2,45) = 7.33, *p* = 0.002), and a significant interaction between factors (*F*(8,180) = 3.99, *p* = 0.004). Simple main effects analysis of the interaction revealed that there was a significant effect of group on bins 3–5 (*F* values > 3.90, *p* values < 0.03), but not on bins 1 and 2 (*F* values < 2.25, *p* values > 0.10). Post-hoc analyses, using the Bonferroni correction, revealed that group Blocking-200 trials showed weaker responding than the Control group on bins 3–5 (*p* values < 0.03), and group Blocking-80 trials on bin 5 (*p* = 0.034)^1^. Group Control and Blocking-80 trials did not significantly differ from one another on any bin (*p* values > 0.08). A similar analysis that included cohort as a factor showed an identical pattern of results and the factor of cohort was not significant and did not significantly interact with other factors.

Responding during the pre-CS period was very low. The Control group responded the lowest, while Group Blocking-200 trials showed the highest level of responding (Control: mean = 0.84 RPM ±0.12 SEM; Blocking-80 trials: mean = 1.07 RPM ±0.17 SEM; Blocking-200 trials: mean = 1.71 ± 0.28 SEM). Analysis of the levels of responding during the pre-CS period revealed a significant effect of group (*F*(2,45) = 4.95, *p* = 0.011). Post-hoc analyses, using the Bonferroni correction, revealed that group Blocking-200 trials showed a higher level of responding than group Control (*p* = 0.012). No other comparisons were significant. Furthermore, when cohort was included as a factor in the analysis the patterns of results stayed the same, and the effect of cohort was not significant and did not significantly interact with other factors.

It is possible that Group Blocking-200 trials showed smaller difference scores than Group Control due to the greater level of pre-CS responding rather than due to impaired conditioning. However, when responding to the blocked cue was assessed independent of pre-CS responses, it was found that there was still a significant group by bin interaction (*F*(8,180) = 3.99, *p* < 0.001), with Group Control showing significantly greater levels of responding than Group Blocking-200 trials in the last two bins (*p* values < 0.02). Therefore, Group Blocking showed weaker conditioned responding as well as greater pre-CS responding in comparison to Group Control.

**Test stage.** Blocking cue. Responding to the blocking cue (A) was similar in all three groups (see Fig. 2, lower panel). There was a significant effect of bin (*F*(4,180) = 27.41, *p* < 0.001), but no significant effect of group (*F* < 1, *p* > 0.80) and no significant interaction of factors (*F* < 1, *p* > 0.90). Responding during the pre-CS interval was very low (overall mean = 0.75 RPM ±0.08 SEM). There was no significant effect of group (*F*(2,45) = 1.46, *p* = 0.24). When cohort was added as a factor to the analysis of the difference scores it was found that mice in the second cohort responded at a higher level to cue A than the mice in the first cohort. However, there was no effect of cohort on pre-CS levels of responding. Cohort did not interact with the effect of group for any measure.

### 3.2 Experiment 2—blocking of a visual cue

**Fig. 3:**
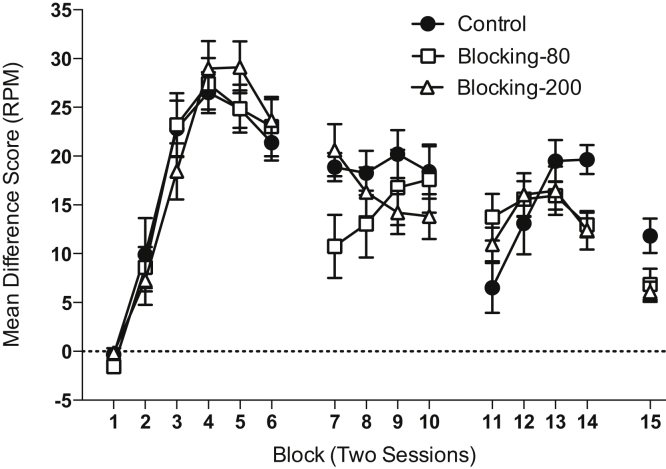
Performance during stages 1 (blocks 1–6), 2 (blocks 7–10) and 3 (blocks 11–15) of Experiment 2. The results of block 15 are separated from those of 11–14 due to the sessions being interleaved between the extinction test sessions. Responding is shown as difference scores in which the rate of magazine entries during the pre-CS period is subtracted from that during the CS. Error bars indicate ±SEM.

**Stage 1.** The results of Stage 1 are shown in Fig. 3. Responding increased over blocks and was similar for all three groups. There was a significant effect of block (*F*(5,145) = 69.25, *p* < 0.001), but no significant effect of group or interactions of factors (*F* values < 1, *p* values > 0.60). Analysis of the pre-CS levels of responding (overall mean = 2.17 RPM ±0.12 SEM) revealed a significant effect of block due to a decline in responding over training (*F*(5,145) = 21.00, *p* < 0.001), but no significant effect of group (*F*(2,29) = 1.94, *p* = 0.16) and no significant interaction between block and group (*F*(10,145) = 1.77, *p* = 0.11).

**Stage 2.** Performance increased over blocks for group Blocking-80 trials that commenced training with cue A and eventually the performance of the three groups converged (see Fig. 3). There was a significant block by group interaction (*F*(6,111) = 2.93, *p* = 0.035), but no significant main effects (*p* values > 0.50). Simple main effects analysis of the interaction revealed that there was a significant effect of group on block 7 (*F*(2,37) = 3.60, *p* = 0.037), but not thereafter (*p* values > 0.50). Post-hoc analyses, using the Bonferroni correction, showed that group Blocking-200 trials responded at a significantly higher level than Blocking-80 trials (*p* = 0.042). There were no other significant differences.

Analysis of pre-CS levels of responding revealed a significant effect of group (Control: mean = 1.25 RPM ±0.22 SEM; Blocking-80 trial: mean = 2.92 RPM ±0.53 SEM; Blocking-200 trials: mean = 1.27 RPM ±0.13 SEM; *F*(2,37) = 6.60, *p* = 0.004), but no significant main effect of block (*F*(3,111) = 2.75, *p* = 0.063), nor interaction of factors (*F* < 1, *p* > 0.70). Post-hoc analyses of the effect of group revealed that group Blocking-80 trials responded at a higher level than both the Control and Blocking-200 trials groups (*p* values < 0.03).

**Stage 3.** Performance increased over blocks for the Control group, for which both cues A and X were novel at the start of compound training (see Fig. 3). There was a reduction in performance in block 15 reflecting extinction of conditioned responding as a consequence of the preceding test sessions. There was a significant effect of block (*F*(4,180) = 23.46, *p* < 0.001), but no significant main effect of group (*F* < 1, *p* > 0.70). However, there was a significant block by group interaction (*F*(8,180) = 5.90, *p* < 0.001). Simple main effects analysis revealed that there was a significant effect of group on blocks 14 and 15 (*F* values > 6.34, *p* values < 0.005) but not on blocks 11–13 (*F* values < 2.65, *p* values > 0.80). Post-hoc analyses, using the Bonferroni correction, revealed that group Control responded at a higher level than groups Blocking-80 trials and Blocking-200 trials on block 14 and higher than Blocking-200 trials on block 15 (*p* values < 0.02). No other comparisons were significant.

Responding during the pre-CS period significantly declined over blocks (*F*(4,180) = 10.42, *p* < 0.001) and significantly differed between groups (Control: mean = 2.55 RPM ±0.40 SEM; Blocking-80 trials: mean = 1.21 RPM ±0.12 SEM; Blocking-200 trials: mean = 0.85 RPM ±0.12 SEM; *F*(2,45) = 12.00, *p* < 0.001), but there was no significant interaction of factors (*F* < 1). Post-hoc analyses of the effect of group, using the Bonferroni correction, revealed that group Control responded at a higher level than groups Blocking-80 trials and Blocking-200 trials (*p* values < 0.005).

**Fig. 4:**
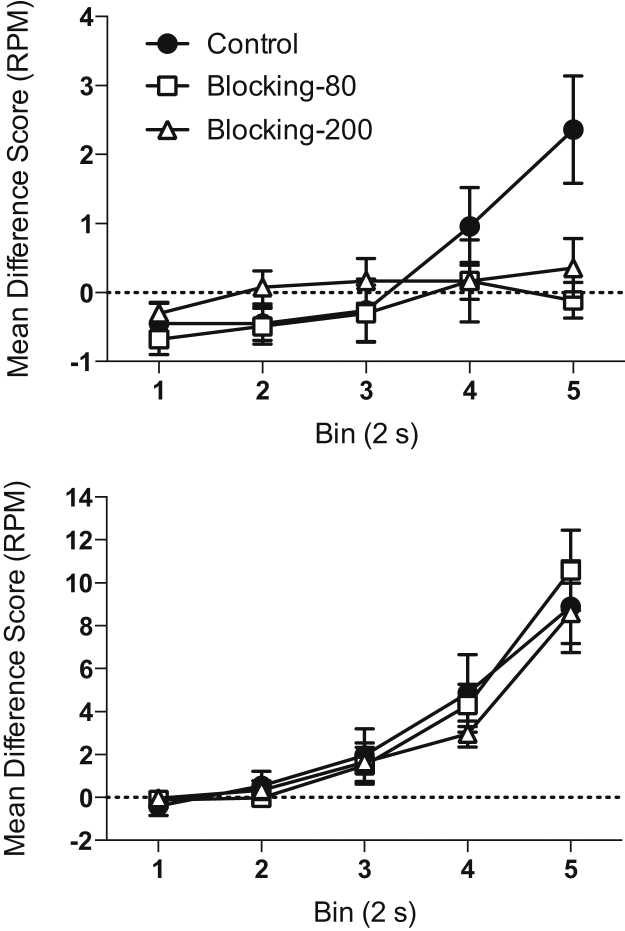
Test performance in Experiment 2. Conditioned responding during the blocked cue (top panel) and blocking cue (lower panel) is shown as difference scores (rate of magazine entries during CS minus the rate of magazine entries during pre-CS period) in five 2 s bins. Error bars indicate ±SEM.

**Test stage.** Blocked cue. The results of the Test Stage are shown in Fig. 4, upper panel. Responding during the presentation of the blocked cue (X) was analysed in five 2-second time bins. Responding showed a marked increase in the final 2 s time bin for the Control group, but not for the other groups. During the first 8 s of the CS responding failed to be above chance (i.e., zero) in all three groups. There was a significant effect of bin (*F*(4,180) = 8.16, *p* < 0.001), but no significant effect of group (*F*(2,45) = 1.81, *p* = 0.18). However, there was a significant interaction between factors (*F*(8,180) = 3.13, *p* = 0.006). Simple main effects analysis revealed that there was a significant effect of group on the last bin (*F*(2,45) = 6.09, *p* = 0.005), but not on the other bins (*F* values < 1.70, *p* values > 0.20). Post-hoc analyses of the effect of group, using the Bonferroni correction, revealed that group Control showed higher responding than the other two groups (*p* values < 0.04), but that there was no difference between the two blocking groups (*p* > 0.9). Whereas there was a significant effect of bin for the Control group (*F*(4,60) = 7.99, *p* = 0.001), there was not for the two blocking groups (*F* values < 1.30, *p* values > 0.30). A similar analysis that included cohort as a factor showed an identical pattern of results, and the effect of cohort was not significant and did not significantly interact with other factors.

Analysis of the pre-CS levels of responding (overall mean = 0.82 RPM ±0.12 SEM) revealed that there was no significant main effect of group (*F*(2,45) = 2.02, *p* = 0.15). A similar analysis that included cohort as a factor showed an identical pattern of results, and the effect of cohort was not significant and did not significantly interact with other factors.

**Test stage.** Blocking cue. Responding increased over the duration of the CS similarly for all three groups (see Fig. 4, lower panel). There was a significant effect of bin (*F*(4,180) = 61.21, *p* < 0.001), but no other significant main effects or interactions (*p* values > 0.70). Analysis of the pre-CS levels of responding (overall mean = 0.78 RPM ±0.09 SEM) was not significant (*F*(2,45) < 1, p > 0.40). A similar analysis that included cohort as a factor showed an identical pattern of results, and the effect of cohort was not significant and did not significantly interact with other factors.

## 4 Discussion

The two experiments reported investigated the effect of prior training with a cue, from one modality, on the ability to block conditioning with another cue from a different modality. The results demonstrate that blocking of appetitive conditioning in mice is dependent on the modality of the blocking cue, and, depending on the particular modality, is dependent on the amount of training with the blocking cue despite the fact that the extent of training did not affect the strength of conditioned responding to the blocking cue. In Experiment 1 a visual cue blocked conditioning of an auditory CS when mice received 200 trials with the blocking cue, but not when mice received only 80 trials. However, in Experiment 2 an auditory cue blocked conditioning of a visual CS regardless of whether mice received 80 or 200 trials. The results demonstrate an asymmetry in the ability of visual and auditory cues to block conditioning of each other, with auditory cues having an advantage over visual cues, and that visual cues need more training in order to block auditory cues.

The differences in visual and auditory cues may ultimately reflect that auditory cues are more salient, or more readily elicit magazine approach behaviour than visual cues, which may instead evoke orienting responses that compete with magazine approach behaviour (Holland, 1977, Holland, 1980a, Holland, 1980b). Indeed, comparison of the performance of the control groups, on the blocked cue extinction test, across the two experiments demonstrates that conditioning was substantially lower for the visual cue (Experiment 2) than for the auditory cue (Experiment 1). However, the effect of trial number on blocking with a visual cue, in Experiment 1, was not simply due to a failure of 80 trials to be sufficient to elicit conditioned responding. By the end of training with the single cue (A) the groups that had received either 80 or 200 trials showed a similar level of conditioned responding. Furthermore, when responding to the blocking cue was tested in extinction in the test phase, the groups did not significantly differ. Therefore, despite a lack of difference between 80 and 200 trials in levels of conditioned responding to the blocking cue, only the group trained for 200 trials showed blocking.

The differences in the effectiveness of 80 or 200 trials on the blocking procedure in Experiment 1 is not due to differences in the experience of the context or the unconditioned stimulus. All groups received identical exposure to these cues. The only difference was the amount of experience with the blocking cue. Furthermore, it is unlikely that the degree of exposure to the context and the unconditioned stimulus influences the blocking effect. In Experiment 2, for one cohort of animals, the amount of exposure to the context and unconditioned stimulus was equated amongst groups, but for another it was not, resulting in the group that received 200 trials having greater exposure to the context and US than the group that received 80 trials. However, the difference in experience of the context and unconditioned stimulus had no significant effect on the extent of blocking.

Although experience of the context was equated between the groups in Experiment 1, it was found that Group Blocking-200 trials showed greater pre-CS levels of responding during the blocked cue test compared with the control group. The high level of pre-CS responding may reflect greater contextual conditioning. However, this effect was not seen during the preceding compound conditioning stage, or in the subsequent blocking cue test. Furthermore, the effect was not found in the two blocking groups in Experiment 2 that both produced significant blocking. Therefore, it is not clear what the cause of increased pre-CS responding might be. Indeed, a study examining another cue competition effect, relative validity, found a result that is the opposite of this effect (Murphy et al., 2001). Thus, rats that received a discrimination between compounds AX+ and BX− (in which A, B and X were discrete cues) showed weaker responding to X and during the pre-CS interval compared to a group that received partial reinforcement of the compounds, AX+/− and BX+/−, suggesting that A and B had reduced learning of X and the contextual cues.

The fact that blocking failed to occur with 80 trials with a visual cue, despite evoking equivalent levels of conditioned responding as when trained with 200 trials suggests that conditioned responding may be a limited measure of the extent of associative learning (see Rescorla and Holland, 1982). Indeed, cues that may be poor at eliciting a particular conditioned response may still be effective in blocking conditioning of that response (Holland, 1977), or may be effective in second-order conditioning of the particular conditioned response (Holland and Rescorla, 1975). In the present study the blocking procedure was effective in demonstrating differences in learning, whereas the strength of conditioned responding was not. Therefore, it is possible that there was no difference in the strength of conditioned responding after 80 and 200 trials because of a possible ceiling effect in magazine approach behaviour with a visual cue.

The results of Experiment 1 potentially contradict a prediction that may be derived from Wagner’s (1981) SOP model of learning. The model proposes that processing of a cue reduces as it comes to be predicted by other stimuli, such as the context in which it is presented. A consequence of this is that overtraining a CS will result in a reduction in the processing that the CS receives, because of further strengthening of the context-CS association. This may then result in an attenuation of blocking due to a failure of the CS to effectively retrieve the representation of the US in the compound-conditioning phase. Therefore, as recently speculated by Jones and Haselgrove (2013), it would be expected that prolonged training of the blocking cue may produce weaker blocking than a blocking cue that has received training that is sufficient only to reach an asymptotic level of associative strength (Jones and Haselgrove, 2013). The hypothesis that a predicted CS is less effective than a CS that is less strongly predicted is supported by a number of findings. First, latent inhibition is context-dependent (Lovibond et al., 1984, Mclaren et al., 1994, Channell and Hall, 1983, Honey and Good, 1993). Therefore, when a stimulus is presented in a context in which it is predicted it acquires conditioned responding less readily than when it is not predicted. Second, a signalled CS evokes weaker conditioned responding than an unsignalled CS (Honey et al., 1993, Mondragon et al., 2003). Third, more frequently experienced CSs receive less attention than less frequently experienced CSs (Jones and Haselgrove, 2013), suggesting that how well the CS is predicted determines the level of processing that it receives. Fourth, in some circumstances CSs that have received prolonged, post-asymptotic training show a decline in conditioned responding (e.g., Schachtman et al., 1987, Pavlov, 1927), which is context-dependent (Bouton et al., 2008, Urcelay et al., 2012).

The results of Experiment 1, in which a visual blocking cue was used, failed to find an attenuation of blocking with prolonged blocking cue training, and instead prolonged training beyond the point that asymptotic levels of conditioned responding was reached was necessary for blocking to occur. Furthermore, the results of Experiment 2, in which an auditory blocking cue was used, also failed to support this prediction. Of course, it may be possible that if mice were trained for a number of trials greater than 200 then an attenuation of blocking may occur, but at the least, the present results suggest that the potential scope for finding such an effect is likely to be limited.

## 5 Conclusion

The results demonstrate that, as for other species, blocking is a robust phenomenon in mice, and that conditioned magazine approach behaviour is determined by cue competition. However, blocking is harder to observe in some circumstances than others. Therefore, although a visual cue may elicit asymptotic levels of conditioned responding, it requires prolonged training in order to be effective as a blocking cue.

## Figures and Tables

**Table 1:** Design of experiment 1.

	Stage 1 (Sessions 1–12)	Stage 2 (Sessions 13–20)	Stage 3 (Sessions 21–28, 31–32)	Test
Blocking (200 trials)	A+	A+	AX+	X
Blocking (80 trials)	B+	A+	AX+	X
Control	B+	B+	AX+	X

Note. Stimulus A was a 10 s presentation of a house light and X was a 10 s presentation a noise. For half of the mice stimulus B was a 10 s clicker and for the other half it was a 10 s presentation of flashing LEDs.

## Footnotes

1 The fact that group differences emerged in the latter portions of the CS duration may suggest that responding by Group Blocking-200 trials was less accurately timed to the occurrence of the US compared to the other groups. To investigate this possibility linear slopes were fitted to the data for each mouse after the responses had been normalized for overall response rates (Jennings and Kirkpatrick, 2006). Analysis of the linear slopes failed to reveal a significant effect of group suggesting that there was no difference in the accuracy of timing. A similar analysis for the results of the blocking test in Experiment 2 also failed to reveal any significant effects.
